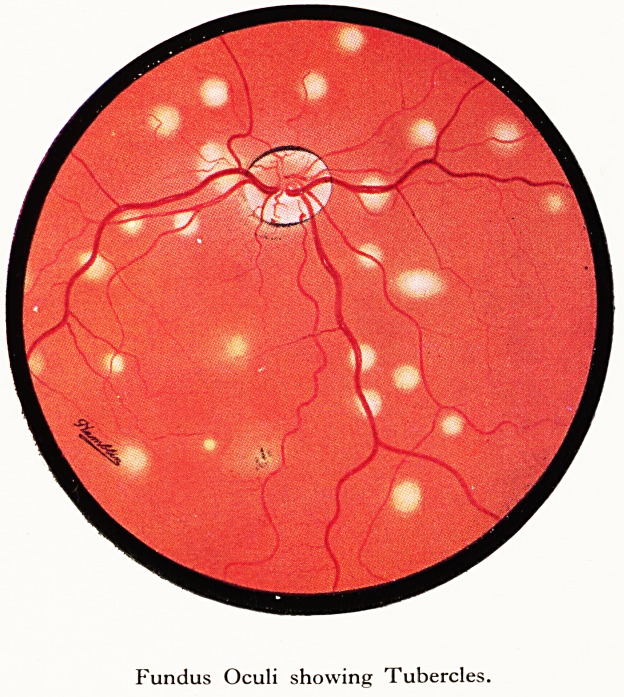# Streptomycin

**Published:** 1951-01

**Authors:** Beryl Corner, Seymour Mason

**Affiliations:** Consultant Paediatrician, United Bristol Hospitals, and Southmead Hospital Group; Medical Registrar, Bristol Royal Infirmary


### STREPTOMYCIN

BY

BERYL CORNER, M.D., M.R.C.P.
*Consultant Paediatrician, United Bristol Hospitals, and Southmead Hospital Group*

AND

SEYMOUR MASON, M.B., B.S., D.C.H.
*Medical Registrar, Bristol Royal Infirmary*

---

In 1944 Waksman in America announced that the actinomyces, streptomyces griseus, produced a powerful antibiotic, streptomycin, which was highly active against various strains of mycobacterium tuberculosis. This drug has been used for the treatment of tuberculosis in this country in research trials organized by the Medical Research Council, from which it is apparent that we have now a valuable antibiotic for use in certain types of tuberculosis.

Streptomycin has, however, some definite limitations. Unfortunately m. tuberculosis tends to develop streptomycin-resistant strains with great rapidity, particularly during treatment of cases of chronic pulmonary tuberculosis, and also infections of the urinary tract. Sufficient emphasis cannot be laid on this point. Not only may the value of the durg in these cases be limited, but the production of drug-resistant strains of bacteria is a potential menace to the rest of the community. Hence great care in selection of cases for treatment is essential and indiscriminate use of the drug must be condemned. However, certain adjuvants, e.g. para-aminosalicylic acid, may delay the development of drug-resistance.

Streptomycin is not absorbed from the alimentary tract and, therefore, it must always be given intramuscularly as well as directly, where possible, into the spaces which it is desired to render free from bacteria. The drug is excreted by the kidney. After intramuscular injection an adequate blood concentration is maintained for six to eight hours and, therefore, the injections need to be given from two to four times in twenty-four hours. Excretion of the drug into the cerebro-spinal fluid is almost negligible, so that meningitis must always be treated by intrathecal injections in addition.

Toxic effects are relatively common. After three weeks of continuous treatment most patients show some evidence of eighth nerve damage—slight vertigo and muscular in co-ordination, which tend to pass off gradually. Some however have persistent difficulty in retaining their balance in the dark; and a great many are left with high-frequency deafness, which may only be detected by audiometry, and is of little clinical significance if the child was talking normally before the onset of the disease. Severe deafness develops in a few cases of meningitis. Particularly when the drug is given intrathecally, marked anorexia is usual early in treatment and may be very troublesome. Renal damage and hypochromic anaemia are much more rare, but regular blood counts and microscopic urine examinations are necessary throughout long courses of treatment. A few patients develop signs of hypersensitivity, morbilliform or urticarial rashes, vomiting, pyrexia and eosinophilia. The drug may also act as a local irritant, if given in too high concentration, particularly into the meninges.

The occasional effects on the administrator must also be mentioned. A very persistent dermatitis of the hands, which may spread to the eyelids or even more extensively, has been seen in a number of nurses. Such subjects tend to develop an extreme hypersensitivity, so that they are unable to continue handling the drug or patients receiving it without an immediate reaction occurring.

The use of this antibiotic in tuberculosis is also limited by certain characteristics of the disease itself. The essential lesion, the tubercle, consists of a collection of inflammatory cells with no blood vessels. As the disease progresses caseation develops, adjacent lesions coalesce, and living tubercle bacilli remain in the centre of necrotic tissue, the blood supply to the infected area being still further reduced by the development of endarteritis obliterans in the surrounding vessels. Thus the blood supply may be insufficient to bring an adequate concentration of streptomycin into contact with the organisms. It is, therefore, not unexpected that, while an acute type of infection, such as miliary tuberculosis, may be responding well to treatment, a focal lesion in the glands or brain may continue to harbour living tubercle bacilli, even after months of treatment; thus providing a constant source of danger until such time as the natural processes of healing may take place. A very long course of therapy may be needed to sterilize all the lesions.

As a result of the research trials it has been shown that streptomycin is of real value in certain types of case, either as a definitive treatment with the object of curing or arresting the disease, or as a prophylactic measure when surgery of a tuberculous lesion is undertaken. The most dramatic results have been achieved in the acute and hitherto lethal forms of the disease, seen particularly in childhood—miliary tuberculosis and tuberculous meningitis. A consideration of the pathology, however, indicates that this drug is useless for the treatment of primary tuberculosis and may even be dangerous, owing to the risk of production of drug resistance.

Many children are infected at some time by the tubercle bacillus, and it has been shown that, in urban areas, 80 per cent. of those reaching early adult life give a positive tuberculin skin reaction (Daniels, 1948). In the vast majority this infection is so minimal that there has been no clinical indication. But under certain conditions, and particularly in very young children, there is ever present the danger of extensive blood dissemination, giving rise to acute miliary tuberculosis: or small silent foci may develop in other organs, particularly the brain. Such foci in the brain may later enlarge, and after rupture into the meninges produce tuberculous meningitis.

The treatment of miliary tuberculosis with streptomycin is now fairly well established. For a period of four to six months the patient should receive continuous daily intramuscular injections of streptomycin: one gramme for children under five years of age and for older patients one to two grammes, according to weight and tolerance. During the first two months of treatment, when it is necessary to maintain a constant high blood concentration, the dose may be divided into four six-hourly injections daily: subsequently it may be given in two twelve-hourly injections. In the majority of cases there is definite clinical improvement after three weeks and clearing of the lung (X-ray) fields may be seen in four to six months. Continuous watch should, however, be kept for the development of silent lesions in the kidney, bones or meninges; provided these complications do not occur, the results of treatment are very good.

Tuberculous meningitis has proved a much more difficult problem. While treatment with streptomycin is a notable advance, this drug alone, administered by the most effective methods yet discovered, is only a partial solution. The prognosis depends very largely on the stage of the disease at which treatment is started, and also probably on the intensity of treatment given. Research trials in this country (including a series of cases treated in the Bristol Children's Hospital Streptomycin Research Unit) have shown that, in early cases where there are no definite physical signs of meningeal irritation, long and intensive courses of combined intrathecal and intramuscular injections result in a 50 to 60 per cent. recovery rate. But if treatment is delayed for only a few days, until definite signs of meningitis are present, the recovery rate falls to 30 per cent. or lower. Cocchi, in Florence, in the largest series of reported cases, shows continued improvement in his results by using much more intensive treatment: and in his more recent cases the recovery rate is much higher, approximating 80 per cent. He adminsters streptomycin by lumbar, cisternal or subdural routes daily for the first two months of treatment, and subsequently on alternate days until the cerebro-spinal fluid has been normal for two months: intramuscular treatment being given daily in addition throughout the whole period. It is clear from our experience in Bristol, as well as published reports, that success in the present state of our knowledge can only be hoped for if these patients are all treated in special units, where medical and nursing staff become skilled in the technique of the treatment, where detailed pathological investigations are readily available and where meticulous care is taken to avoid cessation of treatment or premature discharge from hospital owing to pressure on beds. The pyschological care of these patients during such a long and necessarily unpleasant treatment is a major problem and proper educational facilities, including the service of a " deaf-teacher ", are essential.

To be weighed against all the difficulties involved in treatment of tuberculous meningitis is the overwhelming evidence that patients who recover appear to resume normal health, with very rarely any sequelae other than slight high-frequency deafness. Up to date there is no evidence of any gross deterioration in intelligence. One of our child patients won two prizes in an open newspaper competition for an essay and painting while she was in hospital, still on streptomycin treatment. Another child, who became severely deaf, has been awarded a boarding scholarship at the special grammar school for partially deaf girls. A number of the children are attending ordinary school again, and some of the adults are at work.

It is clear that much work is still required before it can be considered that, for the treatment of tuberculous meningitis, a satisfactory regime has been worked out. It is possible that the combination of streptomycin with other drugs may give better results and it is hoped that further large-scale research will be organized.

The two following cases were treated at Southmead Hospital, and have not been included in the Bristol research trial, although the same programme of investigation and treatment was carried out. They illustrate some of the difficulties that may arise, and one case shows certain features of unusual interest.

**Case 1.** John P., aged three years, was admitted to Cossham's hospital on 27th August, 1949. A previously healthy child with no illnesses, he had been in contact with an aunt who was in a sanatorium with pulmonary tuberculosis at the time of his admission. Three weeks before he had had a cold: this was followed by rapid loss of weight and anorexia, and for three days cyanosis and dyspnoea. On admission he was a severely ill child, very dyspnoeic, cyanosed and wasted: there were scattered râles in both lungs. Radiological appearances were highly suggestive of miliary tuberculosis of the lungs. 29th August, 1949, treatment begun: oxygen tent; intramuscular streptomycin 1.0 gramme daily. One week later, Mantoux 1/100 was negative and E.S.R. 54 mm. in the first hour. 16th September, 1949, condition unchanged, except for some irritability: a number of miliary tubercules were seen in both eye fundi (Plate II), and his spleen was palpable; T. 100.8 deg., P. 128. C.S.F.* 3 lymphocytes, sugar 36 mgm., chlorides 670 mgm., protein 75 mgm., slight

* All figures for C.S.F. are " per c.mm."

PLATE II

Fundus Oculi showing Tubercles.

increase in globulin. In view of these findings, his clinical condition and the general distribution of his disease, it was considered that he was an early case of meningitis. Intrathecal streptomycin therapy was then started, giving 50 mgm. daily for fifty days, followed by fourteen days without intrathecal treatment and then a further course of ten days' intrathecal treatment. Some of the injections were given by cisternal puncture owing to difficulty in obtaining fluid from the lumbar theca. The C.S.F. cell count rose considerably and the protein and sugar values continued to be very abnormal. The diagnosis of tuberculous meningitis was confirmed by the culture of tubercle bacilli from C.S.F.; the stomach washings also yielded tubercle bacilli.

After six weeks' treatment there was evidence of improvement, shown by more stable temperature and fall in E.S.R. By January, 1950, he was a cheerful, very active child, gaining weight well (10 lb. since admission), with a constantly normal temperature and pulse rate. The C.S.F. still showed a marked increase in protein, and a cell count varying weekly from 40 to 120 lymphocytes. The choroid tubercles had completely cleared leaving only very small scars. In February he developed iridocyclitis in the right eye, diagnosed by Mr. Ramsay Garden as tuberculous; vision in that eye became negligible: severe keratitis and conjunctivitis developed in this eye, leaving a scarred cornea. In September, 1950, he is still in hospital, but is very well and has started getting up. Streptomycin treatment was discontinued on May 29th. There has been no pyrexia for six months, and he has gained 19 lb. For eight weeks the C.S.F. has been normal chemically with cells below 7 and the chest X-ray shows complete clearing of the lung fields.

This case is of special interest in the following respects:

(1) The severe picture of disease at the beginning of treatment, with development of meningitis while receiving intramuscular injections.

(2) The large number of choroid tubercles, which cleared completely in five months.

(3) The development of kerato-irido-cyclitis, despite intensive streptomycin treatment for six months.

**Case 2.** Kathleen H. Aged three-and-a-half years, admitted to Southmead Hospital 8th March, 1950. Her only previous illnesses were two attacks of bronchopneumonia and there was no history of tuberculous contact. Five weeks before admission her whole family had had febrile colds, diagnosed as influenza. After this the patient continued to cough and gradually became languid, drowsy and fretful, with poor appetite and occasional vomiting. On admission she appeared an ill child; T. 103 deg., P. 168, R. 45. Her skin was slightly jaundiced, but her principal physical signs were abdominal. The abdomen was distended, and tender, particularly above the umbilicus; the liver was firm and enlarged to the umbilicus, and the spleen was enlarged down to the left iliac fossa; no free fluid or other abnormalities were detected. In the lungs there were scattered coarse râles and a few rhonchi. There were no meningeal signs: but three choroid tubercles were seen. Radiology of the chest showed much enlarged hilar glands with fine mottling in the lung fields, suggestive of miliary tuberculosis. C.S.F. showed a typical rise in cell count and protein, and reduced sugar; tubercle bacilli were cultured. Treatment was begun on the day after admission with combined intrathecal and intramuscular streptomycin.

Her clinical response to treatment was good at first, and after three weeks of treatment her high swinging temperature had settled to normal. Her E.S.R. was 12 mm. in the first hour. Her appetite remained extremely poor; there was no gain in weight and no alteration in abdominal findings. Two months after the onset of treatment she was clearly deteriorating; pyrexia recurred with a daily swing of 100 to 101 deg. There was a tendency for the lumbar theca to block and the C.S.F. showed continually raised protein, up to 320 mgm.; the sugar fell to 22 mgm. and the cell count varied from 30 to 70. The chest X-ray showed considerable clearing of the miliary foci. During the third and fourth months of treatment she was drowsy and very irritable, with occasional vomiting suggestive of raised intracranial pressure. On 20th June she became comatose and died on 22nd June.

Post-mortem examination was performed by Dr. Aidin seven hours after death. The right lung showed no naked eye evidence of tuberculosis. The left lung showed a number of tubercules at the base, a Ghon focus in the upper lobe and a large caseating hilar gland. There were other caseating glands surrounding the trachea and its bifurcation. There was no evidence of tuberculous peritonitis, the spleen was large, firm and dark in colour and the liver appeared normal. A thick gelatinous exudate covered the base of the brain, the Sylvian fissures, and the lumbar region of the spinal cord.

Microscopic findings: very scanty centres of tuberculous granulation tissue in the liver; in the lumbar cord the granulations consisted largely of fibrous tissue; extensive tuberculosis of lungs with areas of caseation; no tuberculosis in the spleen.

This case is of special interest since it shows widespread miliary tuberculosis, including lesions in the liver and meningitis, which responded fairly well to three months of streptomycin treatment. The patient died nevertheless from internal hydrocephalus, due to massive exudate at the base of the brain. The formation of this exudate is one of the major problems in the treatment of tuberculous meningitis, since, despite the bacteriostasis produced and the arrest of the disease processes in the meninges and brain, this mechanical blocking of the cerebro-spinal fluid pathway frequently produces a fatal termination.

REFERENCES

Waksman, S. A. (1944). *Proc. Soc. Exp. Biol.*, N.Y., **55**, 56.

Daniels, M., Ridehalgh, F., and Springett, V. H. (1948). *Tuberculosis in Young Adults, Report on the Prophit Tuberculosis Survey*, 1935–44. 29. London.

Cocchi, C. Exhibition at International Paediatric Congress, Zürich, 1950.

## Figures and Tables

**Figure f1:**